# The Fragility of Statistical Findings in Achilles Tendon Injury Research: A Systematic Review

**DOI:** 10.5435/JAAOSGlobal-D-21-00018

**Published:** 2021-09-02

**Authors:** Robert L. Parisien, Nicholas C. Danford, Ian J. Jarin, Xinning Li, David P. Trofa, J. Turner Vosseller

**Affiliations:** From the Harvard Medical School, Boston, MA (Dr. Parisien); the Columbia University Medical Center, New York, NY (Dr. Danford, Dr. Trofa); the Rutgers Robert Wood Johnson Medical School, New Brunswick, NJ (Jarin); the Boston University Medical Center, Boston, MA (Dr. Li); and the Jacksonville Orthopaedic Institute, Jacksonville, FL (Dr. Vosseller).

## Abstract

Probability analysis with the reporting of *P* values is often used to determine the statistical significance of study findings in the Achilles literature. The purpose of this study was to determine the utility of applying a fragility analysis to comparative trials evaluating Achilles tendon injuries.

**Methods:**

We identified all dichotomous outcome data for comparative studies of Achilles tendon injuries published in 11 orthopaedic journals from 2000 to 2020. The fragility index (FI) was determined by the number of event reversals required to change a *P* value from less than 0.05 to greater than 0.05, or vice-versa. The associated fragility quotient was determined by dividing the FI by the sample size.

**Results:**

Of the 51,357 studies screened, 1,487 met the search criteria, with 51 comparative studies and 177 total outcome events included for analysis. The overall FI was only 4 with an associated fragility quotient of 0.048. One-half of the studies failed to report lost to follow-up data, with an additional 21.6% reporting loss to follow-up of greater than or equal to 4.

**Conclusion:**

Our fragility analysis suggests that Achilles tendon injury outcomes are not as statistically stable as previously thought and should be interpreted with caution.

Evidence-based medicine aids orthopaedic surgeons by providing accurate data of treatment outcomes to properly inform clinical decision-making. To make appropriate data-driven decisions, clinicians must possess a comprehensive understanding of statistical findings.^1^ Researchers often present statistical findings in the form of *P* values, a threshold below which the null hypothesis (H_0_) is rejected in favor of the alternate hypothesis (H_1_). By convention, the value for this threshold is 0.05, which means that the observed difference has a 5% probability of occurring by random chance.^2^ *P* value interpretation is ubiquitous within orthopaedic research and acts as a guide for clinical decision-making through the determination of statistical significance.

The optimal treatment of Achilles tendon ruptures remain controversial because inconclusive findings across the literature have provided more questions than answers with the persistence of a robust debate between nonaurgical and surgical management.^3,4,5,6,7^ Further debate exists regarding the surgical management with some evidence suggesting that minimally invasive repair is superior to open repair, especially about wound complications.^8-10^ As in most orthopaedic literature, *P* values are used to determine the statistical significance of such comparisons. However, the *P* value has received recent scrutiny and criticism, within the academic community, because it may not correlate with clinical significance. For example, the *P* value may be misinterpreted if the data sample from which it is generated contains substantial loss to follow-up, lacks sufficient statistical power, or contains confounding variables.^11,12,13,14^

One way to improve the interpretation of a *P* value is by using the statistical concept of fragility. The fragility of a given statistic is the change in outcome events necessary to alter the overall conclusion drawn from it. When applied to *P* value analysis, the fragility index (FI) provides the investigator with the number of outcome events required to change the value from less than 0.05 to greater than 0.05, thus altering the assessment of its significance. The FI therefore attempts to address the problem with a specific numeric threshold established by convention. The FI was initially proposed by Feinstein in 1990 and has helped inform a body of literature that emphasizes the statistical fragility of findings across various medical disciplines.^15,16,17,18,19,20,21,22^ It has been applied across multiple orthopaedic subspecialties including spine, sport, trauma, and shoulder surgery.^18,23-25^ To further account for the differences in sample size, Ahmed et al^26^ proposed the concept of a fragility quotient (FQ), which is a measure of quantitative significance and is determined by dividing the FI by the sample size. In conjunction with the *P* value analysis, the FI and FQ aid in the interpretation of trial fragility and robustness. As such, studies that possess a low susceptibility to fragility are stronger in their conclusions than studies with high susceptibility to fragility.

The purpose of our study was to determine the statistical fragility of comparative studies in the Achilles tendon injury literature with utilization of FI and FQ analysis. We hypothesized a high susceptibility to fragility within the comparative literature of Achilles tendon injuries.

## Methods

Comparative clinical studies of Achilles tendon rupture management published in 11 prominent orthopaedic journals from 2000 to 2020 were evaluated. These journals consisted of the *Journal of Bone and Joint Surgery, American Journal of Sports Medicine, Orthopaedic Journal of Sports Medicine, Foot & Ankle International, Journal of Foot & Ankle Surgery, Foot and Ankle Surgery, Bone & Joint Journal, Knee Surgery, Sports Traumatology, Arthroscopy, Clinical Orthopaedics and Related Research, Journal of ISAKOS,* and *Journal of the American Academy of Orthopaedic Surgeons*. The journals were selected for their relative prominence within the field of orthopaedic sport medicine and foot and ankle surgery with associated 2019 impact factors listed in Table 1.

**Table 1:** 2019 Impact Factors of Included Journals

Journal	Impact Factor
*American Journal of Sports Medicine*	5.810
*Journal of Bone and Joint Surgery*	4.578
*Clinical Orthopaedics and Related Research*	4.329
*Bone & Joint Journal*	4.306
*Knee Surgery, Sports Traumatology, Arthroscopy*	3.166
*Orthopaedic Journal of Sports Medicine*	2.492
*Foot & Ankle International*	2.292
*Journal of the American Academy of Orthopaedic Surgeons*	2.286
*Foot and Ankle Surgery*	1.776
*Journal of Foot & Ankle Surgery*	1.043
*Journal of ISAKOS*	N/A

2019 journal impact factors obtained from InCites Journal Citation Reports.

The initial search was performed in PubMed with the search criteria “Achilles” and publication date between January 1, 2000, and June 31, 2020. Inclusion criteria were comparative studies reporting dichotomous categorical data and associated *P* values. Exclusion criteria consisted of studies reporting cadaveric data, animal data, in vitro data, nondichotomous data, and those with more than two treatment groups and systematic reviews. The following data from included studies were extracted: first author, journal title, year of publication, the number of study outcomes per group, primary versus secondary outcome, intervention, lost-to-follow up (LTF), *P* value, and the type of study (randomized controlled trial and nonrandomized controlled trial).

**Figure 1:**
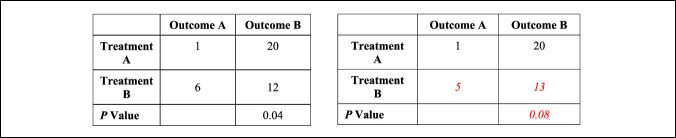
Demonstration of reversal of statistical significance with resultant Fragility Index (FI) = 1

Fragility analysis was performed by manipulating the reported outcome events in a 2 × 2 contingency table until a reversal of significance was determined, with statistical significance defined as a *P* value of less than 0.05. For example, if a particular outcome was initially reported as significant, the number of outcome events required to increase *P* to greater than or equal to 0.05 was determined (Figure 1). If the outcome was initially nonsignificant, the number of outcome events required to decrease *P* to less than 0.05 was determined. The number of altered events required to overturn significance was recorded as the FI for a particular outcome. This was performed for each outcome event with the median value representing the median FI for the entire study. The FQ was calculated for each outcome event by dividing the FI by the corresponding sample size. Interquartile ranges (IQR) for FI and FQ were calculated as the difference between the 25th and 75th percentiles. Fragility analysis was performed for the following subgroups: primary versus secondary outcomes, rerupture, infection/wound complication, return to sport/activity, significant versus nonsignificant outcomes, randomized controlled trial (RCT) versus non-RCTs, studies published in the time periods of 2000 to 2004, 2005 to 2009, 2010 to 2014, and 2015 to 2020 (Table 2).

**Table 2:** Fragility Data Based on Trial and Outcome Characteristics

Characteristic	Events	Fragility Index (Interquartile Range)	Fragility Quotient (Interquartile Range)
All trials	177	4 (2-6)	0.048 (0.021-0.097)
Outcomes			
Primary	17	7 (3.5-10)	0.063 (0.030-0.09)
Secondary	160	4 (2-6)	0.048 (0.017-0.093)
Rerupture	30	3 (2-6)	0.027 (0.010-0.063)
Infection/wound complication	21	3 (2-4)	0.024 (0.013-0.106)
Return to sport/activity	17	5 (3.5-6)	0.078 (0.037-0.112)
Reported *P* value			
*P* < 0.05	32	2.5 (1-5.5)	0.026 (0.014-0.059)
*P* ≥ 0.05	145	4 (3-6)	0.057 (0.025-0.097)
Comparative trial			
RCT	63	4 (2-8)	0.063 (0.033-0.085)
Non-RCT	114	4 (3-6)	0.041 (0.013-0.098)
Year of publication			
2000-2004	15	4 (3-5)	0.042 (0.030-0.097)
2005-2009	71	3 (2-6)	0.038 (0.013-0.122)
2010-2014	25	4 (2-6)	0.058 (0.012-0.010)
2015-2020	66	4 (3-7)	0.062 (0.031-0.085)

## Results

**Figure 2:**
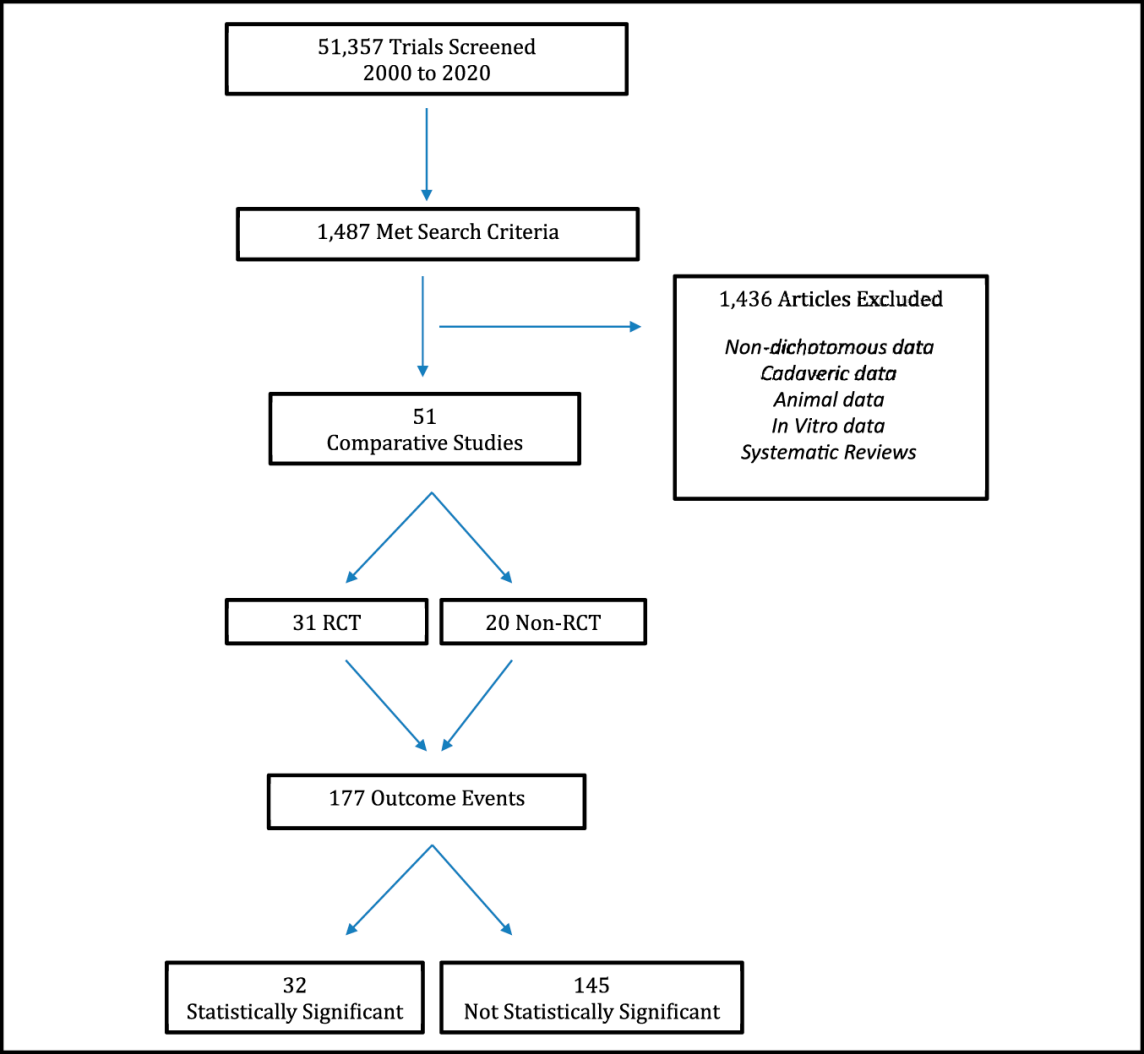
Study identification flowchart

Of the 51,357 studies screened, 1,487 met the search criteria with 51 comparative studies included for analysis (Figure 2). There were 177 total outcome events with 32 initially reported as statistically significant (*P* < 0.05) and 145 initially reported as not statistically significant (*P* ≥ 0.05). Of the 32 outcomes initially reported as statistically significant, the median number of events required to reverse significance (FI) was 2.5 (IQR, 1 to 5.5) (Table 2). The associated FQ for statistically significant outcomes was 0.026 (IQR, 0.014 to 0.059). Of the 145 outcomes initially reported as not statistically significant, the median number of events required to reverse significance (FI) was 4 (IQR, 3 to 6). The associated FQ for initially nonsignificant outcomes was 0.057 (IQR, 0.025 to 0.097). Therefore, statistically significant outcomes were 37.5% more fragile than nonsignificant outcomes. Of the 177 total outcome events, 9.6% (17) consisted of primary outcomes, whereas the remaining 90.4% (160) consisted of secondary outcomes. Primary outcomes were found to be slightly more stable than secondary outcomes, with a FI of 7 (IQR, 3.5 to 10) and 4 (IQR, 2 to 6), respectively. The associated FQ was 0.063 (IQR, 0.030 to 0.09) and 0.048 (IQR, 0.017 to 0.093), respectively. A subanalysis evaluating 30 outcome events relating to tendon rerupture demonstrated a FI of 3 (IQR, 2 to 6) and associated FQ of 0.027 (IQR, 0.010 to 0.063). Accounting for sample size, the FQ for rerupture translates to a reversal of significance through a change of 2.7% of outcome events. Outcomes relating to infection/wound complication (21 events) demonstrated a similar level of fragility with a FI of 3 (IQR, 2 to 4) and FQ of 0.024 (IQR, 0.013 to 0.106). In addition, return to sport/activity outcomes (17 events) demonstrated a FI of 5 (IQR, 3.5 to 6) and FQ of 0.078 (0.037 to 0.112). There was no difference realized in the analysis of comparative trial type with both RCTs and non-RCTs demonstrating an identical FI of four. Further fragility subanalysis per year of publication identified a FI of four from 2000 to 2004, a FI of three from 2005 to 2009, a FI of four from 2010 to 2014, and a FI of four from 2015 to 2020, thus demonstrating consistent statistical fragility over the 20-year period (Table 2).

The overall FI, incorporating 177 outcome events from all 51 comparative studies, was only 4 (IQR, 2 to 6). The overall FQ was 0.048 (IQR, 0.021 to 0.097), indicating the reversal of only 4.8 patients of 100 is required to alter study significance of all included RCTs and non-RCTs when accounting for sample size. Of the 51 included studies, 49% (25) failed to report LTF data with an additional 21.6% (11) reporting LTF of ≥ 4. Therefore, 70.6% (36) of all included studies either did not report LTF data or reported an LTF greater than or equal to the overall FI of 4.

## Discussion

This is the first study to report the FI and FQ for Achilles tendon injury data. Our hypothesis of a high susceptibility to statistical fragility within the literature of Achilles tendon injury was validated. Regardinlg the statistically significant results, a median of only 2.5 outcome events were required to reverse statistical significance. For nonsignificant results, a median of only four outcome events was required to grant a particular result as being statistically significant. Our results therefore reveal the statistical fragility of outcome data for Achilles tendon injuries and the statistical fragility of subgroups including primary and secondary outcomes, rerupture, infection/wound complication, return to sport/activity, significant and nonsignificant outcomes, and outcomes from four distinct time intervals over the course of 20 years. We further demonstrate the critical importance of accurate reporting of LTF data because 70.5% of studies either failed to report their LTF data or reported a LTF of greater than the overall study FI of four, thus suggesting the realization of a reversal of significance by simply maintaining a study follow-up.

*P* value analysis of comparative studies involving Achilles tendon injuries is fragile and therefore needs to be interpreted with caution. Similarly, *P*-values from other subspecialties within orthopaedic surgery have proven fragile as well. In evaluation of 339 outcome events across 102 comparative studies in the sport medicine literature, Parisien et al^24^ demonstrated a FI of five. Fragility analysis by Khan et al^23^ of 48 sport medicine and arthroscopic surgery RCTs revealed a FI of only two. Similarly, in an evaluation of statistical fragility in both the spine and orthopedic oncology literature, a FI of just two was reported.^18,27^ Regarding the orthopaedic trauma literature, Parisien et al^25^ analyzed 198 studies consisting of 775 total outcome events and demonstrated a FI of five and associated FQ of 0.046, representing just 3.8% of the total study population. Furthermore, the evaluation of RCTs in the pediatric orthopaedic literature were found to have a FI of only three.^28^ In addition, in the fragility analysis of 72 clinical trials cited as strong evidence in the *American Academy of Orthopaedic Surgeons* Clinical Practice Guidelines, Checketts et al found a FI of only two with an associated FQ of 0.022. Furthermore, simple application of the Fisher exact test in evaluation of statistical significance nullified significance in 16 (22%) of all included studies, producing a FI of zero. This suggests that the significance of some studies may rest in the particular method of statistical analysis used, representing fragile data.^29^ These previous fragility analyses have applied the concept of the FI to an orthopaedic subspecialty or to data from multiple subspecialties. Our study is unique in that we investigated the statistical fragility of a specific orthopaedic pathology, Achilles tendon injury. We support the use of both FI and FQ when presenting dichotomous results with associated *P* values, especially for studies investigating pathologies whose optimal treatment modalities are not definitively agreed on.

Strengths of this study include the large sample size and comprehensive evaluation of all comparative studies of Achilles tendon injuries over the past 20 years in 11 prominent orthopaedic surgery journals. Our inclusion of both primary and secondary outcomes, rerupture, infection/wound complication, return to sport/activity analysis, significant and nonsignificant *P* values, and non-RCTs, in addition to RCTs, represents additional study strengths as the robustness of such data often influence clinical treatment trends. Although non-RCTs may carry an increased risk of bias and confounding, the inclusion of these trials, in addition to RCTs, provides a more comprehensive evaluation of the existing Achilles tendon injury literature. Furthermore, our inclusion of FQ analysis allows for the interpretation of fragility relative to study sample size. However, utilization of FI analysis has limitations because it applies only to dichotomous data with reported *P* values and cannot be determined for study outcomes reporting continuous data, such as a visual analogue scale or a Likert scale evaluating varying degrees of agreement. Furthermore, given the relative lack of fragility analyses in the peer-reviewed comparative literature, specific fragility thresholds have yet to be determined and remain the focus of future study. In addition, LTF data are often evaluated as a factor contributing to or detracting from study strength. The 49% of studies failing to report LTF data may actually possess minimal LTF; however, we do not know because the authors of those studies failed to report that data. Our reporting of the failure of nearly half the studies to report LTF data is to highlight the lack of standardization in the way in which data are reported in the peer-reviewed Achilles literature. Furthermore, FI does not take into account the time at which events occur.^30^ Nevertheless, the factor of time is less important in surgical trials where success rates of a procedure are of interest but may be more valuable in oncological trials looking at time-to-event endpoints such as increased survival time.^31^

## Conclusion

The *P* value of 0.05 is a well-established cutoff indicating statistical significance, but a similar threshold does not exist for the FI and FQ. Therefore, understanding of how the FI and FQ influences clinical decision-making requires further investigation. Our fragility analysis demonstrates that reported *P* values for Achilles tendon injuries lack statistical stability and should therefore be interpreted in the context of additional data. As such, we recommend the triple reporting of a FI, FQ, and *P* value to provide a comprehensive understanding of the robustness of statistical findings in comparative trials in the Achilles literature.
